# Gastroblastoma in a 5-year-old child: a case report and literature review

**DOI:** 10.3389/fonc.2023.1198762

**Published:** 2023-11-08

**Authors:** Jizhen Feng, Chunxiang Ling, Yingjie Xue, Jiamei Li

**Affiliations:** ^1^ Department of Radiology, Shandong Provincial Hospital Affiliated to Shandong First Medical University, Jinan, Shandong, China; ^2^ Department of Pathology, Shandong Provincial Hospital Affiliated to Shandong First Medical University, Jinan, Shandong, China

**Keywords:** stomach, gastroblastoma, imaging, case report, biphasic tumor

## Abstract

**Background:**

Gastroblastoma is an extremely rare stomach tumor with a biphasic cell morphology of epithelioid and spindle cells. Due to the low incidence rate and the lack of specific clinical characteristics, it is easy to misdiagnose. Detailed imaging analysis is also unavailable. At present, we reported a case of gastroblastoma to analyze its clinical and imaging characteristics. In addition, we reviewed the imaging findings, current diagnosis, treatment, and outcome of gastroblastoma.

**Case presentation:**

A 5-year-old girl was admitted to our hospital with upper abdominal pain and melena. Endoscopic examination showed a protuberant submucosal mass on the greater curvature of the gastric body. Abdominal ultrasonography and an abdominal enhanced computed tomography further confirmed the mass. The patient was pathologically diagnosed with gastroblastoma after radical surgery in February 2021.

**Conclusion:**

We described a rare case of gastroblastoma and may provide a new perspective on imaging diagnosis, treatment, and outcome of this tumor. Gastroblastoma tends to occur in male patients, typically affects young people, and has low malignant potential and a low rate of recurrence and metastasis. Gastroblastoma usually arises in the gastric muscularis propria with hypoecogenic and submucosal characteristics in ultrasound examination and significant enhancement in computed tomography (CT) scan. Surgical resection and regular follow-up after surgery are the main management of the disease. Clinicians should strengthen the understanding of this rare tumor for early detection and treatment.

## Introduction

Gastroblastoma is an extremely rare biphasic gastric tumor characterized by epithelial and spindle cell components, which was first reported by Miettinen et al. (1) in 2009. Gastroblastoma usually occurs in children and young adults (2) but also may occur in the elderly (3). The age range is from 9 to 79 years. To our knowledge, only 20 cases have been reported in the medical literature. Due to the low incidence rate and lack of specific characteristics, it is easy to misdiagnose clinically. Imaging examination preoperative is very important and can provide much useful information for clinical diagnosis. However, a detailed imaging analysis of gastroblastoma is unavailable. We herein reported a case of gastroblastoma in a 5-year-old girl and reviewed the literature to describe in detail its imaging features as well as clinical characteristics.

## Case presentation

A 5-year-old girl visited Shandong Provincial Hospital with upper abdominal pain and melena for 5 days. Two days later, she developed a fever with a maximum temperature of 38.5°C, accompanied by headache and dizziness. The patient had no surgical history, drug allergies, or family history of malignancy. Her laboratory examination showed hemoglobin of 41 g/L.

**Figure 1:**
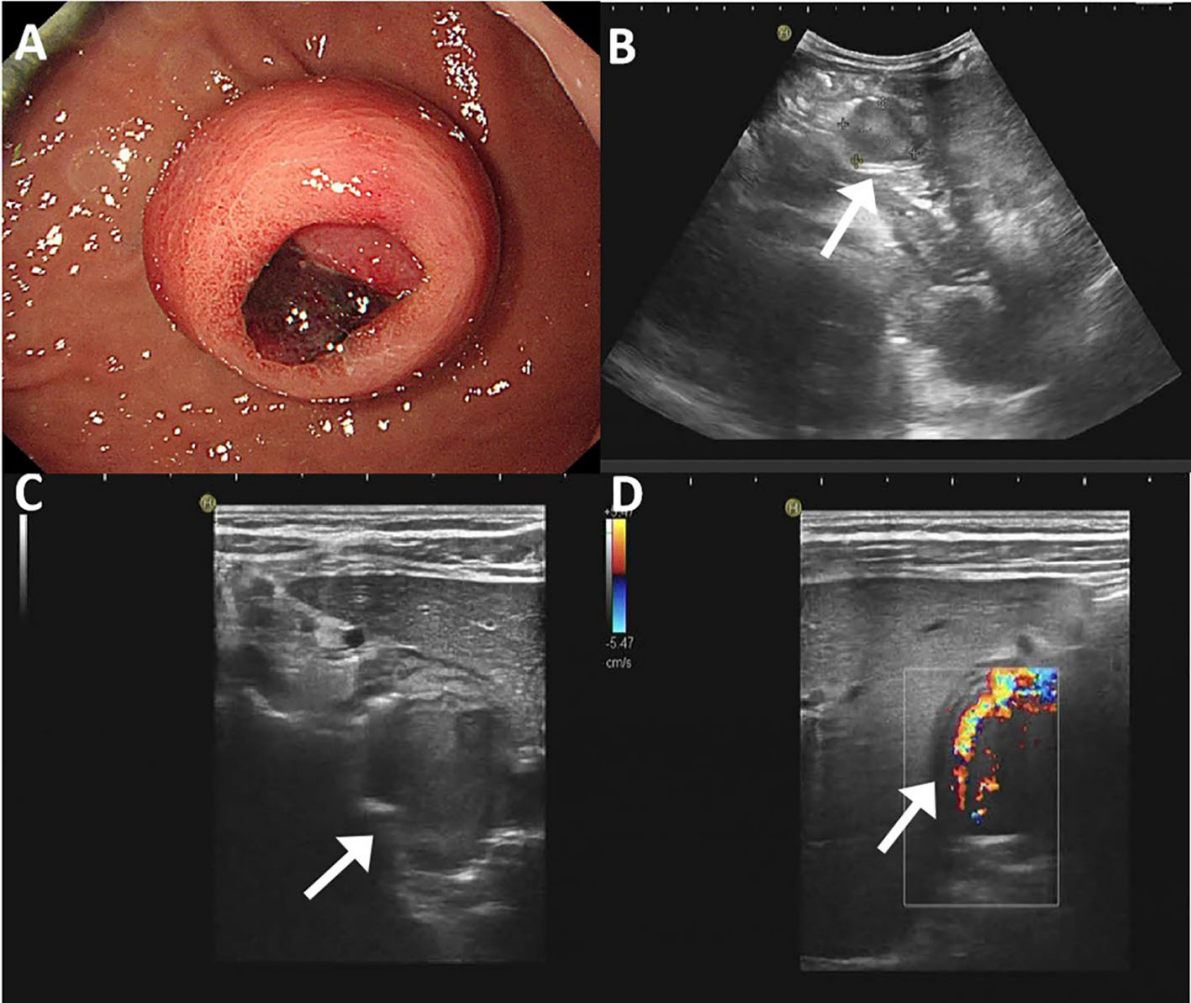
Endoscopic and ultrasonographic features of gastroblastoma. **(A)** A protuberant submucosal mass was detected on the greater curvature of gastric body with ulceration in the center of the mucosal surface. **(B)** A low hypoechoic nodule located in the muscle layer of the gastric wall with clear boundary and regular shape (arrow). **(C)** The tumor protruded into the gastric cavity with uneven internal echo (arrow). **(D)** Color Doppler flow imaging showed strip blood flow signals. Black arrow indicates the tumor (arrow).

**Figure 2:**
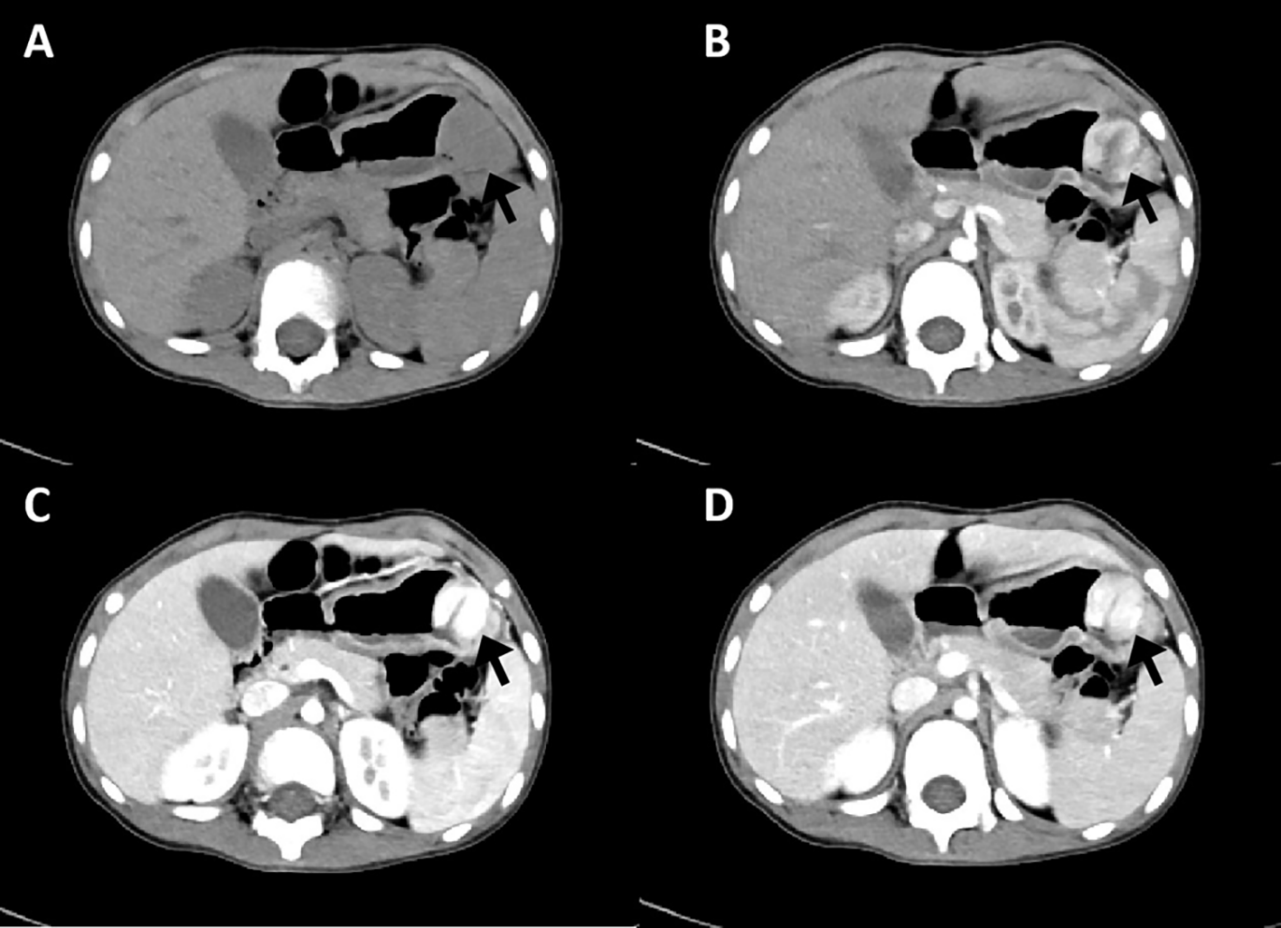
CT imaging features of gastroblastoma. **(A)** CT scan showed a nodule with homogeneous density and well-circumscribed on the lesser curvature of stomach (arrow). **(B)** Obvious and heterogeneous enhancement in arterial phase (arrow). **(C)** Further enhancement in venous phase (arrow). **(D)** Enhancement density of the lesion decreased in delayed phase (arrow).

An endoscopic examination was performed, and a protuberant submucosal mass measuring 3.3 cm × 3.2 cm was detected on the greater curvature of the gastric body with ulceration in the center of the mucosal surface (**Figure 1A**). Abdominal ultrasonography confirmed the mass with a hypoechoic and regular appearance in the gastric wall. A low hypoechoic nodule (2.7 cm × 2.1 cm) was detected in the muscle layer of the gastric wall, with a clear boundary and regular shape (**Figure 1B**). The tumor protruded into the gastric cavity with uneven internal echo (**Figure 1C**). Color Doppler flow imaging (CDFI) showed several strip blood flow signals that could be seen around the nodule (**Figure 1D**). An abdominal enhanced computed tomography (CT) scan showed a 2.2 cm × 2.5 cm nodule with homogeneous density and well-circumscribed on the greater curvature of the stomach, which showed obvious and heterogeneous enhancement in the arterial phase, with further enhancement in the venous phase and decreased density in the delayed phase (**Figure 2**). The tumor was significantly hypervascular, and a glomangioma was suspected. No local invasion or lymph node enlargement was found.

A laparoscopic partial gastrectomy was performed for tumor resection. The patient was placed in a supine position. After anesthesia, the urine tube and stomach tube were inserted for drainage. Approximately 10 ml of coffee-colored old bleeding was extracted from the stomach tube. Then, a laparoscope and 10-mm Trocars were inserted through small incisions in the abdominal wall under general anesthesia. Carbon dioxide pneumoperitoneum was established. Intraoperative observation demonstrated that a solid mass protruded into the stomach cavity with a rich blood supply, central surface necrosis, and bleeding. The tumor was located in the greater curvature and anterior wall of the stomach, approximately 3 cm × 3 cm × 2 cm in size. The operation duration was 2 hours 20 minutes. Intraoperative bleeding was approximately 5 ml. A rapid intraoperative pathological examination was performed, and its result suggested a small round cell tumor, possibly a neuroendocrine tumor. After the operation, the patient’s condition was stable. A total of 100 ml of dark green gastric fluid was drained by gastrointestinal decompression. There were no postoperative complications. The incision had no redness and exudation. The patient was discharged 5 days after surgery. The patient did not receive adjuvant therapy after surgery and continued to receive follow-up every 6 months; there has been no evidence of tumor recurrence 24 months after resection.

**Figure 3:**
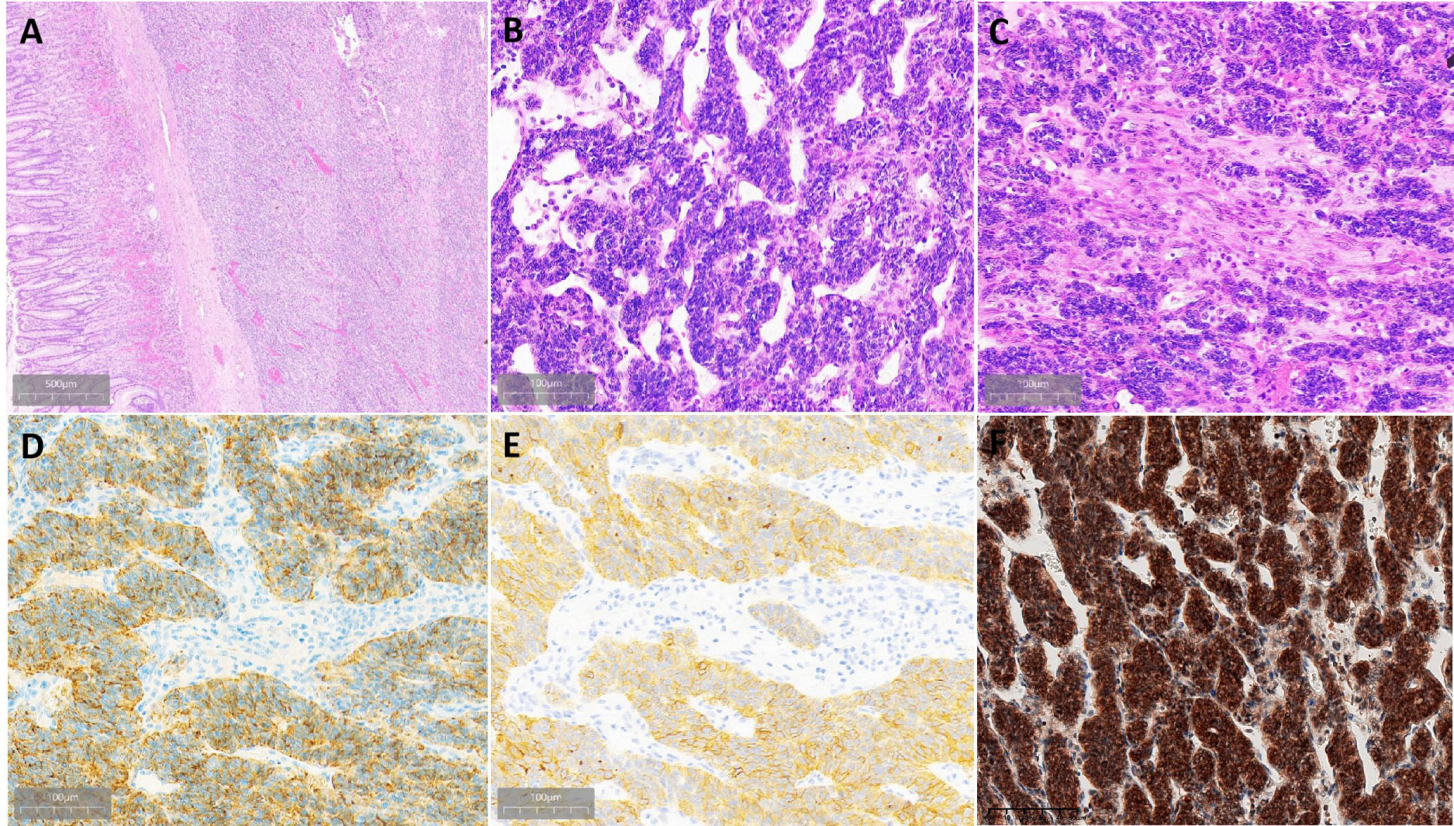
Histopathological findings of gastroblastoma. **(A)** Tumor located in the submucosa and muscularis propria with well-demarcated boundary. **(B)** Epithelial cells are arranged in nests, and the stroma is rich in thin-walled blood vessels. **(C)** Transition of epithelial cells and spindle cells. **(D)** Epithelial cells were strongly and diffusely positive for CAM5.2. **(E)** Part of epithelial cells were strongly positive for CD10. **(F)** Both epithelial cells and spindle cells were strongly nuclear and cytoplasmic positive for Gli1.

Grossly, a well-demarcated tumor was located within the muscularis propria with focal protrusion into the stomach cavity. There was an ulcer on the central surface. The cut surface showed a clear boundary and yellowish-pink with pink-gray. Histologically, the tumor is located in the submucosa and muscularis propria with a well-demarcated boundary (**Figure 3A**). The tumor showed a biphasic morphology, consisting of a large number of epithelial cells and few spindle cells. The epithelial cell component was arranged in nests, sheets, clusters, and cords with small round to oval nuclei with fine chromatin, inconspicuous nucleoli, and scant to moderate eosinophilic cytoplasm. The tumor was rich in cavernous hemangioma-like vascular structures (**Figure 3B**). The spindle cell component was less and with a fascicular growth pattern with pale eosinophilic cytoplasm (**Figure 3C**). The nuclei were elongated or plump with or without small nucleoli. Mitotic figures were infrequent. Necrosis and hemorrhage were found in the tumor.

By immunohistochemistry, both the epithelial and spindle cell components were diffusely positive for vimentin and bcl-2. The epithelial cells also strongly expressed CD56 and CAM5.2 (**Figure 3D**) and showed weak staining for CK and focal staining for CD10 (**Figure 3E**), whereas the spindle cell component lacked expression of CD56 and CAM5.2. Both the epithelial component and spindle cell component were nuclear and cytoplasmic positive for Gli1 (**Figure 3F**). The Ki-67 proliferative index was 30%. DOG1, CD117, SS18-SSX, CD34, SMA, CD99, chromogranin A, synaptophysin, calretinin, S-100, MyoD1, TFE3, HMB45, α-inhibin, STAT6, EMA, SSTR2, and CD31 were negative in both components. The information on antibodies used is shown in **Supplementary Table 1**.

Molecular detection of MALAT1-GLI1 gene fusion is important to confirm gastroblastoma. In the present case, the patient declined to undergo gene detection.

## Discussion

Until the present case, there were only 16 articles reporting 21 gastroblastoma cases. The detailed basic clinical and histological information is summarized in **Table 1**. There appears to be a male predominance, with cases reported in 9 female and 12 male patients to date. Patients’ ages have ranged from 5 to 79 years (mean, 31 years; median, 28 years). Most patients present with abdominal pain, some with melena, bleeding, anemia, dyspepsia, and weight loss. In some cases, a mass lesion is detected by examination (4, 13). Tumors usually arise in the gastric muscularis propria and may extend to the subserosa, submucosa, and mucosa. Ten cases were in the gastric antrum, four were of the body, one was of the fundus (14), one was of the cardia (2), and one was of the pylorus (15), and four cases were not mentioned. The maximum diameters of gastroblastoma have ranged from 0.5 cm to 15 cm (mean, 5.9 cm; median, 5.3 cm). The present case was 5 years old and was the youngest patient until now, and her tumor was 4.5 cm in size and located in the gastric body.

**Table 1:** Clinical Features of Reported Gastroblastomas.

Year	Age/ Sex	Clinic symptoms	Location	Size (mm)	Surgery	Follow up (months)	Recurrence	Metastasis
2009(1)	19/M	Nonspecific abdominal pain	Greater curvature of the gastric body	50×40×25	Subtotal gastrectomy	42	NO	NO
	27/F	Nonspecific abdominal pain	Greater curvature of the gastric body	60×40×35	Partial gastrectomy	60	NO	NO
	30/M	Anemia, fatigue	Gastric antrum	150×120	Partial gastrectomy	168	NO	NO
2010(4)	9/M	Abdominal pain and a periumbilical mass	Gastric antrum	90×65	Resection of the tumor and a segmental resection of gastric antrum and pylorus	93	NO	NO
2012(5)	28/M	Constipation after a motor vehicle accident	Gastric antrum	38× 33×25	a partial gastrectomy	3	NO	Liver
2014(6)	19/F	Diffuse abdominal pain and a mass in the right quadrants of the abdomen	Gastric antrum	105 in diameter	Resection of the tumor and partial distal gastrectomy with 15 lymph nodes resected	20	NO	NO
2014(7)	12/M	More than 3 months of intermittent blood in stool and abdominal mass	Antrum near the lesser curvature	62.3×41.0×37.5	Subtotal gastrectomy and gastroduodenostomy	8	NO	NO
2017(8)	27/M	NA	NA	NA	Resection (not specific described)	12	NO	NO
	28/M	NA	NA	38 in diameter	Resection (not specific described)	NA	NA	Lymph node, liver, peritoneum
	9/M	NA	NA	90 in diameter	Resection (not specific described)	93	NO	NO
	56/F	NA	NA	40 in diameter	Needle Biopsy	NA		Liver
2017(2)	29/F	8 months of epigastric pain and 2 days of hematemesis	Gastric cardia	70×40×40	Atypical partial gastrectomy with splenectomy	6	local regional	Lymph node
2019(9)	43/F	Bleeding	Gastric antrum	53 in diameter	Distal gastrectomy by laparoscopy	100	NO	NO
2019(3)	79/M	Weight loss and dysphagia	Gastric antrum	three masses, 30, 11 and 5	Partial gastrectomy	NA	local regional	NO
2019(10)	53/F	Heartburn and dyspepsia	Curvature near gastric antrum	22.7 × 21.8	Laparoscopic atypical gastrectomy	18	NO	NO
2020(11)	54/F	1 year of epigastric pain and discomfort	Great curvature near gastric antrum	50 × 60	Tumorectomy	14	NO	NO
2021(12)	17/M	3 days of bright red hematemesis and melena	Gastric fundus	63 in diameter	Partial gastrectomy	23	NO	NO
2022(13)	58/M	Annual routine examination	Lesser curvature of the gastric body	24.3 × 14.7	endoscopic submucosal excavation	12	NO	NO
2023(14)	29/F	Upper abdominal pain for over a week	Gastric antrum	70×70×60	Laparoscopic partial gastrectomy	8	NO	NO
2023(15)	26/M	a 2-year history of intermittent abdominal pain	Pylorus	NA	Longitudinal incision through the pylorus	2	NO	NO
2023 (Present)	5/F	Upper abdominal pain and melena	Greater curvature of gastric body	45×22	A laparoscopic partial gastrectomy	24	NO	NO

Gastroblastoma grows in nodules with a solid and sometimes solid-cystic appearance. Histologically, the tumor has biphasic histology, consisting of uniform epithelial cells arranged in nests and uniform spindle cells (16). There are varying proportions of spindle and epithelial cells. The epithelial cells have scant pale cytoplasm, round nuclei, and inconspicuous nucleoli. The spindle cells are long and slender with a fascicular growth pattern. Mitoses are rare in most cases. On immunolabeling, both the epithelial cell component and spindle cell component can express diffuse or focal labeling for CD56 and CD10. The epithelial cell component also can express pan-cytokeratins. The histology of the present case consisted of an overtly epithelioid component and a few spindle cells with similar immunolabeling to reported cases. Because gastroblastoma is biphasic, gastroblastoma should be distinguished from synovial sarcoma. Although the patient declined to undergo further gene detection, tumor cells were SS18-SSX negative and Gli1 positive by immunohistochemical staining. Therefore, the final diagnosis was gastroblastoma.

Considering that gastroblastoma is a rare disease, to the best of our knowledge, there are no detailed reports of its imaging characteristics. The present study evaluated ultrasound and CT imaging results and reviewed the literature. Ultrasound examinations were performed only in three patients; all of the lesions were hypoecogenic and submucosal, two lesions were solid mass, and one was solid-cystic mass. The case in our institution was a hypervascular tumor. CT scan imaging information was available in 12 cases (**Table 2**), including five cases of solid-cystic tumor, two cases of low-density mass, one case of lobulated mass, two cases of mass with on detail, one case of transmural thickening, and one case of heterogeneous tumor. CT enhancement features were reported in six cases, including significant enhancement in two cases and heterogeneous enhancement in four cases. Tumors with clear boundaries were reported in four cases. Tumors appeared exophytic growth toward the lumen in four cases. Gastroblastoma usually arises in the gastric muscularis propria, so it should be differentiated from gastrointestinal stromal tumor (GIST), glomus tumor, and paraganglioma. Among 21 cases of them, GIST was suspected in two cases, glomus tumor in two cases, and paraganglioma in one case. Endoscopic ultrasound was also performed in some cases, which showed submucosal and hypoecogenic mass. Digestive endoscopy examination showed submucosal lesions in two cases. The present case was a submucosal, hypoecogenic, and hypervascular tumor and was misdiagnosed as a glomus tumor.

**Table 2:** CT Features of Reported Gastroblastomas.

Year	CT imaging			
	Mass	Enhancement	Boundary	Growth pattern
2010(4)	Solid and cystic	NA	NA	NA
2012(5)	Heterogeneous	NA	NA	NA
2014(6)	Multiloculated cystic	Septal enhancement	NA	Protrude from the gastric antrum
2014(7)	Mass	NA	NA	NA
2017(2)	Solid-cystic	NA	NA	NA
2019(9)	Mass	Dishomogeneous enhancement	NA	Endoluminal growth
2019(3)	Transmural thickening	NA	NA	NA
2020(11)	Lobulated	Uneven enhancement	Clear borders	Exophytic growth towards the lumen
2022(13)	With a soft tissue density	Significant enhancement	Clear boundary	NA
2023(14)	Multi-cystic	Heterogeneously enhanced	Well-circumscribed	NA
2023(15)	Cystic and solid	NA	NA	NA
2023 (Present)	With low density	Significant enhancement	Clear boundary	Exophytic growth towards the lumen

Surgical resection is the standard treatment for gastroblastoma, including tumorectomy and partial or subtotal gastrectomy by laparotomy or laparoscopy. Endoscopic therapy was performed in one case (13). Additionally, one case received chemotherapy before surgery because of misdiagnosing as adenocarcinoma but was ineffective (5), and one case received postoperative radiotherapy (2). The clinical follow-up varied across different patients, with most of them (81.0%, 17/21) having no evidence of metastasis or recurrence. A total of 18 patients had available follow-up information ranging from 2 to 168 months (mean, 39.2 months; median, 19 months). Liver metastases were reported in two patients at presentation, one of whom also had lymph node and peritoneal spread, and lymph node metastases in one other patient and recurrence after 6 months and only local recurrence in another. One case with local regional has several malignant features at presentation including local invasion, high mitotic activity (21/10 high power field), vascular invasion, and lymph node metastasis, which may be the factors of poor prognosis. The present case had no recurrence after 24 months of follow-up, which indicated that gastroblastoma has an inert biological behavior and a good prognosis.

In conclusion, gastroblastoma tends to occur in male patients, typically affects young people, and has low malignant potential and a low rate of recurrence and metastasis. Gastroblastoma usually arises in the gastric muscularis propria with hypoecogenic and submucosal characteristics in ultrasound examination and significant enhancement in CT scan. Given the rarity of this tumor, the biological behavior is still unclear, and the optimal therapeutic strategy has not been established. More studies are needed to adequately describe the characteristics of gastroblastoma.

## Data availability statement

The original contributions presented in the study are included in the article/**Supplementary Material**. Further inquiries can be directed to the corresponding author.

## Ethics statement

The studies involving humans were approved by Biomedical Research Ethics Committee of Shandong Provincial Hospital. The studies were conducted in accordance with the local legislation and institutional requirements. Written informed consent for participation in this study was provided by the participants’ legal guardians/next of kin. Written informed consent was obtained from the individual(s), and minor(s)’ legal guardian/next of kin, for the publication of any potentially identifiable images or data included in this article.

## Author contributions

JF is a major contributor to writing the manuscript and compiling the figures. CL and YX contributed to the design and format of figures and tables. JL confirmed the pathological findings. JF and JL designed, organized, and helped revise the manuscript. All authors contributed to the article and approved the submitted version.
