# Supplementary material for: Gastroblastoma in a 5-year-old child: a case report and literature review
Source: Front Oncol. 2023 Nov 8;13:1198762. doi: 10.3389/fonc.2023.1198762 (PMC10666783; doi:10.3389/fonc.2023.1198762)
Supplement: Supplementary file 1 [file Table_1.docx]

Supplementary Table 1. Information of antibodies

| Antigen | Clone | Manufacturer | Dilution |
| --- | --- | --- | --- |
| α-inhibin | AMY82 | Zhongshan Golden Bridge Bio-technology | Ready-to-use |
| bcl-2 | OTIR1H2 | Zhongshan Golden Bridge Bio-technology | Ready-to-use |
| CAM5.2 | OTI1B12 | Zhongshan Golden Bridge Bio-technology | Ready-to-use |
| CD10 | OTI1C9 | Zhongshan Golden Bridge Bio-technology | Ready-to-use |
| CD31 | UMAB30 | Zhongshan Golden Bridge Bio-technology | Ready-to-use |
| CD34 | 10C9 | Zhongshan Golden Bridge Bio-technology | Ready-to-use |
| CK | AE1/AE3 | Zhongshan Golden Bridge Bio-technology | Ready-to-use |
| CD117 | EP10 | Zhongshan Golden Bridge Bio-technology | Ready-to-use |
| CD56 | UMAB83 | Zhongshan Golden Bridge Bio-technology | Ready-to-use |
| CD99 | PCB1 | Zhongshan Golden Bridge Bio-technology | Ready-to-use |
| calretinin | OTI1D5 | Zhongshan Golden Bridge Bio-technology | Ready-to-use |
| chromogranin A | LK2H10 | Zhongshan Golden Bridge Bio-technology | Ready-to-use |
| DOG1 | OTI1C6 | Zhongshan Golden Bridge Bio-technology | Ready-to-use |
| EMA | LK2H10 | Zhongshan Golden Bridge Bio-technology | Ready-to-use |
| Gli1 | OTI2D5E2 | Origene | 1:100 |
| HMB45 | HMB45 | Zhongshan Golden Bridge Bio-technology | Ready-to-use |
| Ki-67 | UMAB107 | Zhongshan Golden Bridge Bio-technology | Ready-to-use |
| MyoD1 | EP212 | Zhongshan Golden Bridge Bio-technology | Ready-to-use |
| synaptophysin | OTI1C9 | Zhongshan Golden Bridge Bio-technology | Ready-to-use |
| SMA | UMAB237 | Zhongshan Golden Bridge Bio-technology | Ready-to-use |
| SSTR2 | EP149 | Zhongshan Golden Bridge Bio-technology | Ready-to-use |
| STAT6 | EP325 | Zhongshan Golden Bridge Bio-technology | Ready-to-use |
| S-100 | 15E2E2+4C4.9 | Zhongshan Golden Bridge Bio-technology | Ready-to-use |
| SS18-SSX | E9X9V | Zhongshan Golden Bridge Bio-technology | Ready-to-use |
| TFE3 | EP285 | Zhongshan Golden Bridge Bio-technology | Ready-to-use |
| vimentin | EP21 | Zhongshan Golden Bridge Bio-technology | Ready-to-use |
